# Cutaneous and Labyrinthine Tolerance of Bioactive Glass S53P4 in Mastoid and Epitympanic Obliteration Surgery: Prospective Clinical Study

**DOI:** 10.1155/2015/242319

**Published:** 2015-10-04

**Authors:** Daniele Bernardeschi, Yann Nguyen, Francesca Yoshie Russo, Isabelle Mosnier, Evelyne Ferrary, Olivier Sterkers

**Affiliations:** ^1^AP-HP, Groupe Hospitalier Pitié-Salpêtrière, Service d'Oto-Rhino-Laryngologie, Département d'Otologie, Implants Auditifs et Chirurgie de Base du Crâne, 75013 Paris, France; ^2^Sorbonne Universités, UPMC Univ Paris 06, 75005 Paris, France; ^3^Inserm UMR-S 1159, “Réhabilitation Chirurgicale Mini-Invasive et Robotisée de l'Audition”, 75018 Paris, France

## Abstract

*Objective*. To evaluate the cutaneous and the inner ear tolerance of bioactive glass S53P4 when used in the mastoid and epitympanic obliteration for chronic otitis surgery. *Material and Methods*. Forty-one cases have been included in this prospective study. Cutaneous tolerance was clinically evaluated 1 week, 1 month, and 3 months after surgery with a physical examination of the retroauricular and external auditory canal (EAC) skin and the presence of otalgia; the inner ear tolerance was assessed by bone-conduction hearing threshold 1 day after surgery and by the presence of vertigo or imbalance. *Results*. All surgeries but 1 were uneventful: all patients maintained the preoperative bone-conduction hearing threshold except for one case in which the round window membrane was opened during the dissection of the cholesteatoma in the hypotympanum and this led to a dead ear. No dizziness or vertigo was reported. Three months after surgery, healing was achieved in all cases with a healthy painless skin. No cases of revision surgery for removal of the granules occurred in this study. *Conclusion*. The bioactive glass S53P4 is a well-tolerated biomaterial for primary or revision chronic otitis surgery, as shown by the local skin reaction which lasted less than 3 months and by the absence of labyrinthine complications.

## 1. Introduction

Bioactive glass S53P4 (BG) is a bioactive material that elicits a specific biological response at the interface of material and tissue resulting in the formation of a bond between them [1]. It is a silica-based biomaterial with bone bonding properties [2, 3], osteoconductive and osteoproductive in promoting migration, replication, and differentiation of osteogenic cells [4, 5]. The material is a mixture of oxides composed by 53% SiO_2_, 23% Na_2_O, 20% CaO, and 4% P_2_O_5_. After the interaction with body fluids, the rapid ions exchange forms a Si-rich layer that interacts with Ca^2+^ and PO_4_^3−^ to allow the crystallization of hydroxyl carbonate apatite over the surface of the granules [6]. Together with this chemical mechanism, a cellular mechanism promotes osteostimulation and new bone formation: the bioactive glass stimulates the growth and maturation of osteoblasts and promotes the maintenance of osteoblastic phenotype [7, 8] that produces the bone matrix guided by the hydroxyapatite layer formed over the granule's surface. Studies in vivo demonstrated that when implanted in a cavity created in the tibia of rats in contact with bone marrow, it promotes the new bone formation and, 8 weeks after implantation, animals implanted with BG had 50% more bone formation than the control group [9, 10].

Furthermore, the BG has the unique property of being antibacterial over many aerobic and anaerobic bacteria [11, 12]. The inhibition of bacterial growth is probably due to the release of ions at the first stage of implantation which causes elevation of the pH and of the osmotic pressure. Moreover, the BG can suppress* Staphylococcus aureus* and* Pseudomonas aeruginosa* biofilms formation on titanium alloy disc in vitro [13, 14].

Clinical studies regarding the use of BG have been published in orthopedic (trauma [15, 16], benign bone tumors [17], and chronic osteomyelitis [18, 19] surgeries) and craniofacial (frontal [20, 21], maxillary [22] sinus surgery) fields.

Few reports have been published in ear surgery concerning the rehabilitation of canal-wall-down mastoidectomies [23–25]. Unlike long bones, the mastoid bone contains cells in contact with air. The mastoid cavities are covered by modified respiratory epithelium with ciliated and secretory cells and the bone does not contain bone marrow. Moreover, the mastoid cavities are in direct contact with the inner ear that contains auditory and vestibular hair cells whose functioning is dependent of inner ear fluids ionic composition [26–28].

The aim of this prospective observational uncontrolled study was to assess the cutaneous (retroauricular and the external auditory canal) and inner ear tolerance of the BG S53P4 when used for obliteration of canal-wall-down (CWD) and canal-wall-up (CWU) mastoidectomies.

## 2. Material and Methods

This study, carried out between May 2013 and January 2015, was authorized by the ethical institutional board, and all patients gave their written consents for the use of their personal clinical data. The bioactive glass S53P4 is produced by BonAlive Biomaterials Ltd. (Turku, Finland) and has been approved for clinical use in 2004 in Europe and in 2007 in the United States.

The tolerance of the material was clinically evaluated for the skin of the retroauricular and external auditory canal (EAC) 1 week, 1 month, and 3 months after surgery with physical examination and otoscopy under microscope. The presence of pain was also evaluated during the same examination. The presence of otorrhea and infection was also investigated.

Inner ear tolerance was evaluated clinically with the presence of vertigo and/or dizziness and with bone-conductive pure-tone audiometry performed one day after surgery.

Forty-one cases (39 patients, two operated bilaterally) were included: there were 22 males and 17 females. The mean age was 46 ± 15 years (mean ± SD, range 16–79 years). There were 25 right side and 16 left side cases. The mean preoperative bone-conduction hearing threshold calculated at 0.5, 1, 2, and 3 kHz was 32 ± 17.2 dB.

**Figure 1:**
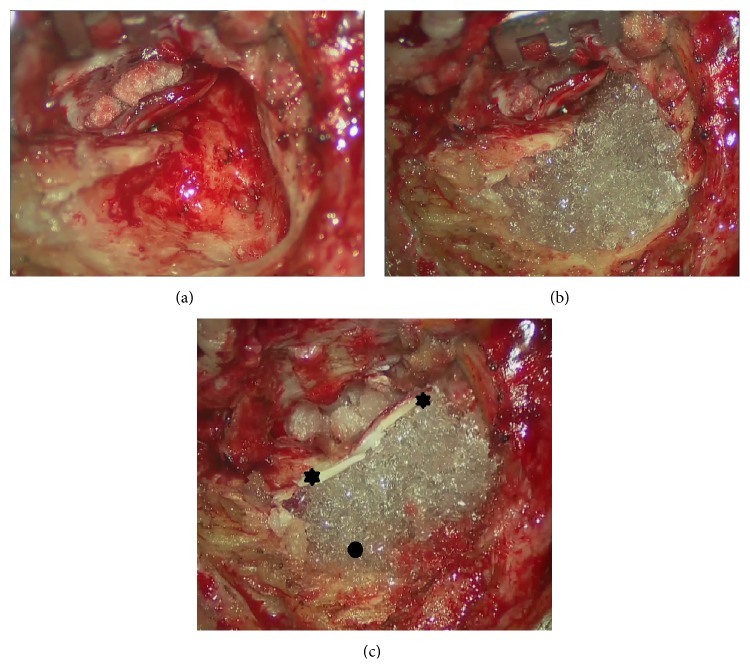
Surgical view of left ear after reconstruction of the external auditory canal and mastoid obliteration with bioactive glass granules. (a) After reconstruction of middle ear in a canal-wall-down mastoidectomy; (b) after positioning of the granules; (c) at the end of the procedure before skin closure: note the mastoid completely obliterated by the granules (black oval) covered by cartilage (black stars).

All patients were operated on general anesthesia with facial nerve monitoring system NIM-Response 2 and NIM-Response 3 (Medtronic, Jacksonville, FL, USA). After bacteriological sampling collected with a swab in the EAC and/or in the mastoid cavity, a retroauricular skin incision and a C-shaped muscoloperiosteal flap were performed. Fibrous tissue (temporalis fascia, retroauricular fibrous tissue, perichondrium, and pericranium) was harvested. Cartilages from cymba, cavum conchae, and tragus were sampled and thinned using* Precise Cartilage Knife* (Kurz, Tuebingen, Germany). Afterwards primary (*n* = 10) or revision (*n* = 25) CWD or CWU (*n* = 6) mastoidectomy was performed. A cholesteatoma was found in 23 cases. After the removal of the lesion, the reconstruction started with the middle ear (tympanic drum grafting and ossiculoplasty) and was followed by mastoid and epitympanic obliteration using the BG granules (size 0.5/08 mm) previously moistened with 3 cc of saline solution. The granules were carefully covered by cartilage and fibrous tissue in the reconstructed EAC (Figure 1), and particular attention was taken to ensure that the reconstructed EAC reached the level of the lateral skin of the meatoplasty. Then, the reconstructed EAC was filled with MeroGel ear packing (Medtronic, Jacksonville, FL, USA).

Perioperative antibioprophylaxis with amoxicillin/clavulanate was performed in all patients and it was continued until it has been adapted to the results of the perioperative bacteriological test. The treatment was delivered for 14 days following recommendations for chronic otitis [29] and cochlear implant surgeries [30] with mastoid obliteration. Ear drops of ofloxacin were administered to all patients for 1 month.

## 3. Results

All the mastoid cavities and epitympanic spaces were filled with a volume of granules between 4 and 5 cc. In all the cases, cartilage and fibrous tissue were enough to allow a complete covering of the exposed granules in the EAC even in multioperated ears; there was no need to perform a muscoloperiostal flap to cover the granules.

Results of bacteriological test are shown in Table 1. In 23 cases (56%) bacteria or fungus was found, sometimes in association (*n* = 5).

All but 1 patient conserved the preoperative bone-conductive hearing threshold; this patient had an opening of the round window membrane during the dissection of the cholesteatoma in the hypotympanum and experienced ipsilateral total hearing loss postoperatively. Contrariwise, three cases of preoperative lateral semicircular canal fistulas, where an opening of the posterior labyrinth occurred during surgery, maintained the preoperative bone-conduction hearing threshold. Postoperative bone-conductive hearing threshold was 28 ± 18.4 dB (*n* = 40, deaf patient excluded).

Seven days after surgery, the retroauricular skin was healthy in all patients but 1; the latter experienced retroauricular swelling without inflammation of the skin and moderate aseptic otorrhea; this patient was treated conservatively.

Five patients (12%) complained of otalgia without any sign of inflammation. No vertigo or dizziness was reported by patients.

One month after surgery, retroauricular skin was healthy in all patients. Skin of the EAC showed some degree of inflammation with swelling of the posterior wall in 13 cases (32%). All cases were successfully treated with the positioning of an ear pop wick and administration of ear drops (association of antibiotics and corticosteroid) for 14 days. One patient experienced purulent otorrhea due to* Pseudomonas aeruginosa* infection that was successfully treated with intravenous ceftazidime for 14 days. One patient presented uncovered granules in the EAC due to cartilage resorption: this patient underwent revision surgery under local anesthesia to cover the exposed granules in the EAC two months after the primary surgery.

**Figure 2:**
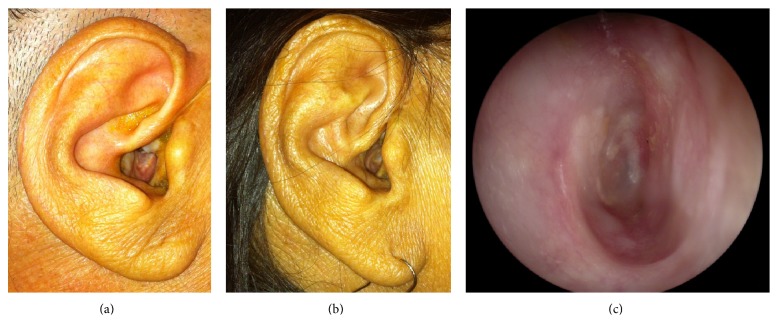
Preoperative (a) and postoperative (b) physical examination and postoperative otoscopy (c) of a patient operated for the obliteration of a canal-wall-down mastoidectomy with BonAlive granules, 3 months after surgery.

Three months after surgery, healing was achieved in all patients. No cases of retroauricular or EAC skin inflammation were reported, with a healthy skin in the retroauricular and EAC examination (Figure 2).

## 4. Discussion

The use of biocompatible materials has been reported for many years in otologic surgery. In this field, especially in revision surgeries and/or in multioperated ears, the availability of autologous materials could be a challenge for the otologic surgeon. Moreover, the possibility of the donor-site morbidity and the risk of resorption over time should be kept in mind when choosing them for reconstruction. This is why a lot of reports in the literature described the use of biocompatible materials either in ossicular chain replacement prosthesis and/or mastoid obliteration, with excellent results.

Regarding mastoid obliteration, either autologous materials (muscular flap [31], bone [32], bone pate [33], cartilage [34], and fat [35]) or biocompatible materials (silicon blocks [36], hydroxyapatite cement [37], and titanium posterior wall prosthesis [38]) have been already described.

The BG is a bone-substitute material that allows the restoration of bone stock by resorption and further apposition of new bone from a differentiated tissue.

In the orthopedic field, other bone-substitute materials such as bioresorbable bioactive ceramics (calcium phosphates, hydroxyapatite, and tricalcium phosphate) are most commonly used as osteoconductive bone graft substitute [39, 40]. The main differences between these bone substitutes have already been described [6].

In ear surgery, the use of biphasic ceramic (BC), a mixture of hydroxyapatite and tricalcium phosphate with fibrin sealant, has been described by some authors for the rehabilitation of CWD mastoidectomies [29, 41–43]. However, some cases of infection of the implanted material and subsequent revision surgery in order to remove the granules have been reported.

We used BC as an obliterating material for the obliteration of the mastoid and epitympanic cavities from 2006 to 2013 in 130 cases. Our results on the first 59 cases with a minimum follow-up of 1 year were retrospectively reviewed [29]. The same surgical technique as well as the same antibiotherapy was applied in this prospective study using the BG. From a surgical point of view, the BG is easier to manage than BC with fibrin sealant, since with the latter positioning of the granules in the mastoid cavity should be performed within 10 minutes following the preparation. Furthermore, the granules of BC are larger than the granules of BG and this makes the obliteration of smaller spaces (as e.g., the anterior epitympanum) more difficult.

Regarding the cutaneous tolerance, in our series, BC granules were removed because of infection or pain in 4 patients. These complications occurred early in the postoperative period. Conversely, no case of revision surgery for removal of granules with the use of BG has been observed in the present study. With the surgical technique, the surgeons, and the antibiotherapy being the same in the two studies, this difference might be due to the antibacterial property of the BG: indeed, the results of our preoperative bacteriological tests showed that all the bacteria we found were sensitive to the BG mechanism of action in vitro [11, 12, 14]. Moreover, even in case of fungus infection, we did not observe any infection of the implanted material even if the BG properties have not been tested yet against fungus in vitro.

Furthermore, we wondered about the inner ear tolerance of the BG. Because of the increase of pH and osmotic pressure in the hour following the placement of the granules, it was proved that this material does not have adverse side effects on the labyrinthine structures. All but one patient maintained the preoperative bone-conduction threshold, and no patient complained of vertigo even in case of opening of the posterior labyrinth during the dissection of the cholesteatoma. The only patient that experienced immediate postoperative sensorineural hearing loss underwent the opening of the round window membrane during the surgery, and hence the hearing loss can be attributed to the surgical procedure rather than an inner ear toxicity.

The first report on the use of BG S53P4 BonAlive in ear surgery was realized by Stoor et al. [23]; they retrospectively reviewed 7 patients treated for mastoid obliteration with BG and they focused only on the size of postoperative cavities without any concern about the inner ear tolerance. Similarly, Silvola [24], in a prospective study carried out on 14 patients, did not observe any infection of the granules and described a good skin tolerance. But Sarin et al. [25], on a retrospective study on 26 patients treated over a 25-year period, experienced 2 cases of recurrent postoperative otorrhea, 1 case of opened wound, 1 case of exposed BG granules in the EAC, and 1 case of profound deafness, but no statement was made concerning the cause of this hearing loss.

In the present study, we did not observe any adverse reaction to BG granules; only one patient had to be reoperated because there were uncovered BG granules in the EAC one month after surgery. However, this also occurred with the use of BC in a larger percentage of patients, suggesting an incomplete recovering of the granules rather than an adverse effect of the biomaterial. In our opinion, attentive coverage of the granules with cartilage is essential and mandatory. As already reported [24], fibrous tissue is not enough and this could expose the implanted material to infection and/or extrusion.

In conclusion, this prospective study focused on the tolerance of BG demonstrated that the BG S53P4 is a very well-tolerated material for mastoid and epitympanic obliteration. Anatomical and functional results need to be evaluated in a longer follow-up period.

## Figures and Tables

**Table 1:** Results of perioperative bacteriological test (*n* = 41).

BACTERIA	*N*
Aseptic	18
*Staphylococcus *	8
(i) *aureus* (*n* = 4)	
(ii) Coagulase negative (*n* = 2)	
(iii) Epidermidis (*n* = 1)	
(iv) Association (*n* = 1)	
*Pseudomonas aeruginosa *	3
*Candida *	2
(i) *albicans* (*n* = 1)	
(ii) *parapsilosis* (*n* = 1)	
*Streptococcussanguinis *	1
*Stenotrophomonas maltophilia *	1
*Propionibacterium* sp.	1
*Turicella otitidis *	1
*Proteus mirabilis *	1
*Aspergillus niger *	1
Association of bacteria	4
